# Transformation and Management of Long-Bone Atypical Cartilaginous Tumours

**DOI:** 10.3390/cancers17193178

**Published:** 2025-09-30

**Authors:** Edmund Coke, Ofir Ben-Gal, Ashish Mahendra, Julian Pietrzycki, Sarah Vaughan, Sanjay Gupta

**Affiliations:** Glasgow Royal Infirmary, Glasgow G4 0SF, UK; edmund.coke@gmail.com (E.C.); ofir.bengal@sheba.health.gov.il (O.B.-G.); ashish.mahendra@nhs.scot (A.M.); julian.pietrzycki@mater.org.au (J.P.); sarah.vaughan@nhs.scot (S.V.)

**Keywords:** atypical cartilaginous tumour (ACT), enchondroma, chondrosarcoma, central cartilaginous tumour, follow-up study

## Abstract

**Background/Objectives**: Atypical cartilaginous tumours (ACTs) are intermediate, locally aggressive chondroid tumours in the appendicular skeleton. Due to the potential for transformation into high-grade chondrosarcomas, management typically consists of regular MRI follow-up and, occasionally, surgery. We primarily aimed to examine the rate of malignant transformation in ACTs in our hospital; secondarily, we aimed to identify the factors influencing management choices and outcomes. **Methods**: All patients referred between 2013 and 2020 with a long-bone ACT were identified from the unit database. For this retrospective study, we analysed the imaging, management, and outcomes for the patients discussed at our musculoskeletal radiological conference. **Results**: A total of 59 patients were included; of these, 0 cases of malignant transformation were observed with a mean follow-up time of 8.4 years. Of the presenting cases, the musculoskeletal radiological conference advised that 6 should be biopsied, 40 should receive MRI follow-up, 7 should receive X-ray follow-up, and 6 should be re-examined in clinic. Subsequently, 12 patients underwent surgery due to continued pain, diagnostic uncertainty, and historical practices. Of these, seven experienced continued post-operative pain. **Conclusions**: None of the encountered ACTs underwent malignant transformation, supporting previous findings that this transformation is a rare phenomenon. Furthermore, of the small sample of patients undergoing surgery, less than half were left pain-free. These findings support a more conservative approach to ACT management, with the potential to discharge after an initial review.

## Simple Summary

Atypical cartilaginous tumours (ACTs) are long-bone chondroid tumours that are classified as intermediate in nature, between benign enchondromas and malignant chondrosarcomas. They are locally aggressive, but their management is primarily based around an understanding that ACTs have the potential to undergo malignant transformation into chondrosarcomas; however, the actual rate of these transformations remains unclear. In the past, curettage was the mainstay of treatment, but a more conservative approach of watchful waiting, utilising repeat imaging, is increasingly being employed. This retrospective study aimed to examine the rate of malignant transformation observed in ACT cases referred into our Scottish Tertiary Centre. Of the 59 patients referred, no instances of malignant transformation were observed. In the context of other studies showing similarly low rates of malignant transformation, our findings could support the avoidance of long-term follow-up in favour of discharging with appropriate safety-netting procedures.

## 1. Introduction

Under the 2020 WHO classification, atypical cartilaginous tumours (ACTs) are defined as intermediate, locally aggressive chondroid tumours of the appendicular skeleton [1]. This places them on the spectrum of central cartilage tumours, between benign enchondromas and malignant chondrosarcomas. It can often be hard to differentiate ACTs from enchondromas. Radiologically, they are both lobulated lytic lesions; however, ACTs are typically larger, with more pronounced endosteal scalloping [2]. Histologically, they both predominantly contain hyaline cartilage; ACTs sometimes also have a small amount of myxoid cartilage [3]. They are differentiated from their axial equivalents, grade 1 chondrosarcomas (CS1s), due to the fact that CS1s have poorer clinical outcomes [4] and the ability to metastasise [5]. Despite this, ACTs still have the potential to undergo malignant transformation into high-grade chondrosarcomas [6].

ACTs are currently a topic of great importance due to their increasing incidence and continued uncertainty surrounding optimal management approaches. Between 1989 and 1996, ACT/CS1 incidence was 1.20 per million people per year, but by 2005–2013, it had risen to 6.63 per million people per year; this increase was perhaps driven by the ageing nature of the population, as well as the increased use of imaging [7]. They can occur as incidentalomas, with a prevalence of 0.4% being reported (compared to the 2.8% for enchondromas) [8]. The management options for ACTs remain varied, with a recent consensus meeting identifying it as “possibly the most controversial area”, requiring “balancing the morbidity of treatment with the requirement for oncological safety” [9]. Active surveillance with radiological follow-up is commonly implemented [10], but curettage (often with local adjuvant therapies) is also employed [11]. This uncertainty in management is likely due to some confusion persisting over the malignant transformation rates of ACTs. Historically, rates of around 5% have been reported [5], but more recently, the risk has been placed at <1% [12].

The goal of this study was primarily to assess the rate of malignant transformation of ACTs at the Glasgow Royal Infirmary (Glasgow, UK) and to determine whether there are any predictive factors. Secondarily, we aim to evaluate the factors that influenced management choices.

## 2. Materials and Methods

**Figure 1:**
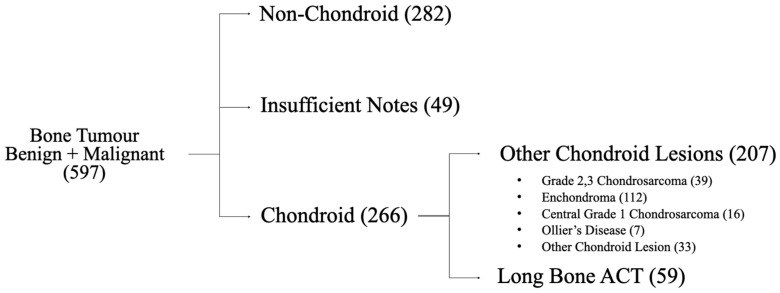
Flowchart of selection of long-bone ACTs from bone tumour database.

Data gathering was performed as part of an audit, for which approval was obtained from the Caldicott guardian. All patients with long-bone ACTs were identified from a prospectively maintained database of bone tumours at the Glasgow Royal Infirmary (see Figure 1). Patients were initially identified in August 2022 and then followed up through a review of notes, with the final review occurring in March 2024, giving a minimum of 18 months follow-up. Participants were adult patients who had been diagnosed between January 2013 and December 2020 through discussion at the musculoskeletal (MSK) radiological conference (which includes at least one MSK radiologist and one orthopaedic oncology fellowship-trained surgeon). Patient inclusion was not dependent on positive biopsy results but based on the MSK radiological conference interpretation of radiological appearance (including features such as tumour size, lysis, scalloping, and cortical breach) and clinical features. Patients were included in the study if they had a long-bone lesion that the MSK radiological conference diagnosed as either (i) an ACT, (ii) a CS1, or (iii) some form of low-grade chondroid tumour that was concerning enough to necessitate follow-up. The latter criterion ensures that, in the retrospective analysis, patients were not missed due to the use of historic nomenclature (e.g., chondroid tumour of uncertain malignant potential (CLUMP) or grade 0.5 chondrosarcoma [13]) or the absence of an explicit diagnosis. It should be noted that, during this time period, simple enchondromas were discharged by the conference, leaving the lesions selected for follow-up as those of a more concerning nature. Patients were excluded from the study if they had axial CS1s or acral and flat bone ACTs. In addition, we excluded patients with a history of Ollier’s Disease or Maffucci Syndrome, as chondroid lesions in these patients are known to behave more aggressively [14,15].

Patients’ clinical presentations were recorded, including presence of pain, nature of pain, and presence of other pathology. The location of the lesion and a decision on whether the pain could be deemed incidental (i.e., found on imaging due to another pathology or pain attributable to another pathology) was also noted. MRIs were analysed in an unblinded manner by a clinical fellow in orthopaedic oncology (OBG) for features previously found to be associated with higher-grade tumours [14]. These were size, scalloping, cortical breach, presence of a soft tissue mass, or lysis. Data from the MSK radiological conference were recorded, including date, diagnosis, and management recommendation. Biopsies were recommended if there was sufficient uncertainty around the diagnosis based on radiological and clinical presentation to warrant further investigation. Similarly, both biopsy data (if performed, date, outcome) and surgical data (if performed, date, type, indication) were also recorded. Current indications for surgery are persistent pain over the ACT site or continued uncertainty in diagnosis, but in some older cases, surgery was performed as part of routine ACT management at the time. Finally, outcomes were examined, namely the presence of malignant transformation, recurrence, and presence of post-operative pain.

## 3. Results

There were 59 patients included in this study. There were over twice as many women in the dataset as men, with a mean age of 50.8 years at the time of the MSK radiological conference diagnosis (see Table 1). The mean follow-up time was 8.4 years. Anatomically, 27 of the lesions were situated in the femur (7 proximally, 19 distally, and 1 mid) and 18 sat in the proximal humerus. The remaining 14 were situated in the smaller long bones (6 in each of the fibula and the tibia and 1 in each of the ulna and radius).

Clinically, 88% presented with pain. Of those experiencing pain, 73% experienced pain on activity, 12% experienced pain at rest, and 15% experienced both (see Table 2). Of those experiencing pain, 86% had some underlying musculoskeletal pathology other than ACT to which the pain could be attributed. Underlying pathologies included the following: osteoarthritis (29%), tendinopathy (19%), trauma (17%), and meniscal tears (10%). Of the seven patients that lacked any pathology other than the ACT, four were still explicitly deemed to be incidental at the MSK radiological conference, leaving three patients with pain that was solely attributable to the ACT and not deemed to be incidental. Of those not presenting with pain (12%), other cancer-related imaging accounted for the majority.

On the analysis of the MRIs for scalloping, the following observations were made: 31 had no scalloping, 7 had some scalloping but did not meet the cutoff, 14 had scalloping across >⅔ of their width, and 7 had scalloping across >⅔ of their width and length. Moreover, two of the lesions had signs suggestive of a cortical breach, and seven had changes suggestive of lysis, but none had signs of a soft tissue mass. Lesions had a mean size of 43.8 mm (±3.1 mm (standard error of the mean)). Of the lesions, 30 had diameters larger than 40 mm and 21 lesions had diameters larger than 50 mm, with 2 lesions being over 100 mm (see Table 3).

The MSK radiological conference advised that 6 patients should be immediately biopsied with a view to conducting surgery, 40 should receive MRI follow-up, 7 should have X-ray follow-up, and 6 needed further examination in a clinic. Radiological and clinical features were analysed to determine whether they could predict management choice, but a significant association was only found between larger mean size and immediate biopsy (*p* < 0.05 as determined by one-way analysis of variance).

In terms of further management, both biopsies and surgeries were carried out. There were 6 further biopsies, bringing the total number of biopsies to 12. Based on these biopsies, the lesions were diagnosed as follows: two were diagnosed as enchondromas, five were diagnosed as ACTs, four were diagnosed as enchondromas/ACTs, and one was deemed to be inconclusive. Twelve patients underwent procedures; of these, eleven were curetted and one underwent radio frequency ablation (the inconclusive biopsy). Indications for surgery included the uncertainty of the diagnosis (four lesions), historical management protocol of CS1 (four lesions), and persistent pain over the ACT region (four lesions).

With respect to the outcomes, there was no evidence of transformation into higher-grade chondroid lesions. There were four deaths, none of which were attributed to chondroid lesions. Post-operatively, there was one case of recurrence, and seven patients had continued pain.

## 4. Discussion

Epidemiologically, our findings are consistent with the current knowledge: the mean age at diagnosis (50.4 years) and the overrepresentation of women in the sample concur with previous findings for ACTs and chondroid lesions [7,16,17].

With respect to our primary goal, this retrospective study of long-bone ACTs found no cases of malignant transformation amongst the 59 cases reviewed. This highlights the rarity of this phenomenon and is in keeping with the findings of other recent studies (see Table 4). The lack of transformations observed despite the presence of radiological signs on MRI, that have previously been reported as being associated with higher-grade chondroid tumours (e.g., scalloping, size and lysis), suggests that such features are not indicative of malignant transformation risk. Deckers et al. came to a similar conclusion, noting that, for enchondromas and ACTs, “scalloping does not predict natural course” [10]; previous results suggest that scalloping can be exhibited by benign lesions [18].

The optimal management of ACTs remains unclear. In our study, there were a range of management options recommended by the MSK radiology conference, predominantly MRI. The fact that lesion size was correlated with an initial biopsy is unsurprising given that, historically, this has been associated with malignant potential [3]. However, the lack of any other correlations between features generally associated with high-grade tumours and management decisions likely reflects the fact that the parameters extracted in this study failed to capture the whole picture that the conference sees, as well as possible shifts in practice over time. The rate of conversion (11%) from initial conservative management to surgical management is very similar to that identified in previous studies [19,20], as is the role played by pain as both an indication for surgical intervention and a post-operative complication.

The complexity of ACT management was addressed to some degree by Patel et al. in developing the Birmingham Atypical Cartilage Tumour Imaging Protocol (BACTIP) [21], which provides a protocol for how enchondromas and ACTs can be managed after initial MRI. BACTIP uses size, endosteal scalloping, and aggressive features to categorise central cartilage tumours with BACTIP categories I and II. These categories are intended to contain enchondromas and ACTs, while higher-grade chondrosarcomas should fall into category III. However, this protocol is appropriate for when lesions are first encountered by non-specialist radiologists in the community, with the main focus being on referring concerning cases on to tertiary centres. It also places a lot of emphasis on MRI follow-up for up to 3 years, but this is based on the aggressiveness of the initial appearance and not the risk of transformation. At the end of the initial validation paper [12], they reflect that “the hawkish observer might query, with long-term malignant transformation rate of <1% in the present series, whether the follow-up advocated by BACTIP is actually justified”; but they state that the protocol is “a reasonable adverse compromise”.

There are numerous other papers reporting outcomes from ACT follow-up. A scoping review of Pubmed on 11th May 2025 using the search terms “Atypical Cartilag* Tumo*r*”) OR (“Cartilag* Lesion* of Unknown Malignant Potential”) OR (“Grade 1 Chondrosarcoma*”) OR (“Grade I Chondrosarcoma*”) OR (“Chondrosarcoma* Grade 1”) OR (“Chondrosarcoma* Grade I”) OR (“Low-Grade Chondrosarcoma*”) returned 635 papers; of these, 11 are studies in which passive management had been employed. One was excluded, as its dataset was reused in a later paper [12]; a further paper, that by Kumar et al. 2016 [22], was added after being identified during the course of further research. These papers, seen in Table 4, propose a variety of management suggestions for ACTs. While some do base their recommendations on perceived proxies for malignant transformation, such as growth or other aggressive features, none are actually based around the key reason for follow-up imaging, which is detecting malignant transformation. Out of 1584 patients followed up with over 67 months, on average, only 5 cases of malignant transformation were noted. However, there are some caveats when looking at these papers together. The key point to note is that the vast majority of these studies involve mixed-lesion cohorts, where enchondromas are included with ACTs; this means that the transformation rate is not an accurate reflection of what might be expected for cohorts containing ACTs alone. Furthermore, in many of the studies, certain patients would either undergo surgery immediately or during the follow-up period, with factors such as pain or aggressive radiological features influencing this treatment decision, leaving behind lesions that are not representative of ACTs in general. Heterogeneity in study design also makes inter-study comparison hard. Taken together, these factors mean that, from the current literature, an accurate rate for malignant transformation of ACTs cannot be determined.

**Table 4:** Studies examining rate of ACT transformation.

Author	Date	Lesion Types	Patient Numbers	Mean Follow-Up (Months)	Transformation
Deckers et al. [20]	2016	Enchondroma + ACT	41	66	0
Kumar et al. [22]	2016	Enchondroma + ACT	46	60	0
Chung et al. [23]	2018	Enchondroma + ACT	21	45	0
Omlor et al. [24]	2019	Enchondroma + ACT	153	88	0
Deckers et al. [10]	2021	Enchondroma + ACT	128	50	0
LaPrade et al. [19]	2023	Enchondroma + ACT	57	55	0
Woltsche et al. [25]	2024	Enchondroma + ACT	176	27 *	0
Scholte et al. [26]	2024	ACT	117	41	0
Van Den Berghe et al. [27]	2024	BACTIP Category I + II	61	38	0
Davies et al. [28]	2025	BACTIP Category I + II	721	86	4
Opper et al. [29]	2025	BACTIP Category I + II	63	35	1

* Median follow-up.

A complete understanding of the risks of ACT transformation is key as it dictates the way in which these tumours are managed. Understandably, doctors are keen to pick up malignant tumours early to maximise patient outcomes and avoid litigation; however, this must be balanced with the cost of active observation, biopsies, and surgery at both patient and health service levels. From the patient’s perspective, although active surveillance does not reduce their quality of life compared to their baseline at 6 months [30], there is still the possibility that longer-term follow-up might generate anxiety (“scanxiety”) and logistical difficulties. This was highlighted in the context of chondrosarcoma follow-up in a recent consensus meeting [9]. Furthermore, the biopsies and surgical procedures that can result from follow-up also carry risks of complication [11,26]. There must be a point at which the risk of developing a malignancy is so low that the balance between help and harm tips towards the latter. Similarly, at the level of a healthcare system, there comes a point at which the time, personnel, and cost of active monitoring and management is not justified by the chance of detecting or preventing a very small number of transformations. For example, while there has not been a cost analysis specifically on ACT follow-up, an American study looking at the cost of enchondroma surveillance found that the median post-referral cost for enchondromas was USD 10,244.11 (broken down into USD 1914.93 for clinics, USD 1506.98 for X-rays, and USD 6501.95 for MRIs) [31]. In our own hospital, the cost of a consultant-led clinical appointment is calculated to be GBP 233 and that of an MRI is calculated to be GBP 130. Given these costs, it could be argued that, taken as a whole, the risk of malignant transformation in ACTs is so low, and the drawbacks of long-term follow-up imaging are so substantial, that safety-netting a patient to watch out for red flags for malignant transformation is sufficient and appropriate. The key red flag symptom to watch out for is pain. This is a symptom associated with higher-grade chondrosarcomas, and so it can be indicative of malignant transformation [32]. As such, new or increased pain is already being used to safety-net patients between follow-up imaging [10]. This less-intrusive approach is supported by recent findings showing that there is no significant difference in survival among patients with ACTs, regardless of whether they underwent operative management or not [33].

In terms of the limitations of this study, the sample size was small, making it hard to detect the <1% transformation rate currently hypothesised in the literature. Secondly, the follow-up time, stretching from diagnosis to March 2024, was variable, and may have been too short to capture malignant transformation events [34]. It is also important to note that 11 patients underwent curettage before the end of the follow-up period. Although the risk of malignant transformation was not explicitly an indication for these procedures, it may be that they represent a population that is more likely to transform and potentially could have done so if left unoperated on. This would bias the reported rate of malignant transformation to be artificially low. Another problem inherent to studying ACTs is that the accurate identification of the lesions is a struggle in itself. Radiological and histological diagnosis is often inconsistent with large interobserver variation seen in both [35]. This might be part of the reason why, of the 12 biopsies performed, histologically, 2 were diagnosed as enchondromas and 4 were diagnosed as enchondromas/ACTs. In the future, radiological diagnosis is likely to improve through utilizing machine learning models with groups distinguishing enchondromas from ACTs [36,37,38] and ACTs from high-grade chondrosarcomas [39].

## 5. Conclusions

The exact rate of ACT transformation remains unclear, but what is evident from this study and the other literature is that it is exceedingly rare. Thus, consideration should be given as to whether ACTs require long-term follow-up if the detection of malignant transformation is the primary goal. A pragmatic approach might be to discharge patients diagnosed at tertiary centres with safety-netting advice relating to changes in pain, with a 6-month MRI to catch misdiagnosed chondrosarcomas. However, given the small sample size and retrospective nature of this study, a randomised controlled trial would be useful to establish the optimal management strategy. There are also some caveats when looking at the literature with the key point being that the vast majority of these studies involve mixed-lesion cohorts, where enchondromas are included with ACTs, making the transformation rate an inaccurate reflection of what might be expected for cohorts containing ACTs alone. 

## Figures and Tables

**Table 1:** Patient demographics of patients reviewed by the MSK radiological conference from 2013 to 2020.

Gender	
Female	40 (68%)
Male	19 (32%)
Age	
Mean	50.8 year
ACT Location	
Femur	27
Humerus	18
Tibia	6
Fibula	6
Ulna	1
Radius	1

**Table 2:** Clinical presentations of patients.

Pain	
Total	52
At Rest	6
On Activity	38
Both (Activity and Rest)	8
Pain: Alternative Pathology	
Osteoarthritis	15 *
Tendinopathy	10
Trauma	9
Meniscal Tears	5
Pathological Fracture	2
Post-Fracture Imaging	2
Inflammatory Arthritis	1
Epiphysiodesis	1
Hereditary Multiple Exostosis	1
Iliotibial Band Syndrome	1
Cervical Radiculopathy	1
Shoulder Dysplasia	1
None	7
No Pain	
Total	7
Cancer-Related Imaging	4
Post-Fracture Imaging	1
Unclear Indication for Imaging	2

* Of which 3 had concomitant meniscal tears and 1 had concomitant tendinopathy.

**Table 3:** Radiological analysis.

Scalloping	
Absent	31
<2/3 Width and Length	7
>2/3 Width	14
>2/3 Width and Length	7
Cortical Breach	
Yes	2
No	57
Soft Tissue Mass	
Yes	0
No	59
Lysis	
Yes	7
No	52

## Data Availability

The original contributions presented in this study are included in the article. Further inquiries can be directed to the corresponding authors.

## Abbreviations

The following abbreviations are used in this manuscript:

ACT	atypical cartilaginous tumour
CS1	grade 1 chondrosarcoma
MSK	musculoskeletal
CLUMP	Chondroid Tumour of Uncertain Malignant Potential
BACTIP	Birmingham Atypical Cartilage Tumour Imaging Protocol
